# Expression of the First Recombinant Anti-Tumoral Snake Venom Kunitz-Type Serine Protease Inhibitor

**DOI:** 10.3390/toxins14030170

**Published:** 2022-02-25

**Authors:** Maram Morjen, Wassim Moslah, Imen Touihri-Baraketi, Najet Srairi-Abid, José Luis, Naziha Marrakchi, Jed Jebali

**Affiliations:** 1 Laboratory of Biomolecules, Venoms and Theranostic Applications, LR20IPT01, Pasteur Institute of Tunis, University of Tunis El Manar, Tunis 1002, Tunisia; maram.morjen@yahoo.fr (M.M.); wassim.moslah@gmail.com (W.M.); najet.abidsrairi@pasteur.tn (N.S.-A.); naziha.marrakchi@pasteur.tn (N.M.); 2 Laboratoire des Substances Naturelles, Institut National de Recherche et d’Analyse Physico-Chimique (INRAP), Sidi Thabet, Ariana 2020, Tunisia; imen.touihri@gmail.com; 3 CNRS-UMR 7051, Institut de Neuro Physiopathologie (INP), Université Aix-Marseille, 27 Bd Jean Moulin, 13385 Marseille, France; jose.luis@univ-amu.fr; 4 Medicine School of Tunis, University of Tunis El Manar, 15 Djebel Lakhdhar, Street La Rabta, Tunis 1007, Tunisia

**Keywords:** snake venom, Kunitz-type serine protease inhibitor, recombinant PIVL, tumorogenesis

## Abstract

PIVL is a Kunitz-type serine protease inhibitor that was previously characterized from Tunisian snake venom, *Macrovipera lebetina transmediterranea*. It reduced glioblastoma cells’ development and significantly blocked angiogenesis in in-vitro and ex-vivo models. PIVL exerted these effects by interfering with αvβ3 integrin. In order to produce a biological active recombinant, the cDNA cloning and expression of PIVL was performed in *Escherichia coli* (BL21)-DE3 cells using pET-22b (+) vector. The recombinant PIVL protein (rPIVL) was purified by nickel affinity chromatography and has recognized monoclonal anti-His antibody. Functionally, rPIVL exhibited potent anti-tumor cell effects as well as anti-angiogenesis properties. Interestingly, we found that both native PIVL (nPIVL) and rPIVL modulated PI3/AKT and MAPK signaling pathways. In all, our results showed that we have successfully expressed the first active anti-oncogenic snake venom Kunitz-type protease inhibitor that can be a potential therapeutic drug against glioblastoma, in its native or recombinant form.

## 1. Introduction

Glioblastoma is the most frequent type of malignant brain tumor in humans. The worst patient prognosis is caused by a highly aggressive cell growth, angiogenesis, rapid migration, and infiltration of anaplastic cells [1]. The classic therapy involves surgical resection, radiation and chemotherapy by oral administration of temozolomide [2]. However, the ability of tumor cells to invade adjacent brain tissue prevents complete surgical resection of glioblastoma, leading to recidivism in patients [3,4]. Moreover, the main problem of chemotherapeutic agents is the lack of selectivity towards tumor cells. These drugs induce toxicity and alter healthy tissue during treatment. Thus, recent research studies focus on improving new generations of biopharmaceutical components that increase selectivity by targeting specific molecules to treat cancer [4]. Snake venoms are known to be a source of therapeutic agents. Indeed, some drugs derived from snake venom are approved for treatment of thrombosis and hypertension, such as Integrilin^®^ (Eptifibatide), Captopril^®^ (Enalapril), and Aggrastat^®^ (Tirofiban). Moreover, other molecules are considered as promising therapeutic candidates for preclinical or clinical applications [5].

Kunitz-type inhibitors represent a class of small proteins commonly found in such venoms. These proteins from *Viperidae* and *Elapidae* snake venoms contain in their sequence about 60 amino acids with three disulphide bridge-connected cysteine residues at sites C1–C6, C2–C4, and C3–C5 [6], called Kunitz-type domain. It has been reported that peptides with Kunitz-domain are involved in a variety of physiological and pathological processes, including fibrinolysis, coagulation, and inflammation [7]. Previous studies showed that Kunitz-type protease inhibitor isolated from *Bungarus multicinctus* snake venom reduced human neuroblastoma SK-N-SH cell migration and invasion [8]. In our previous work, we purified a Kunitz-type serine protease inhibitor from the venom of the Tunisian snake *Macrovipera lebetina transmediterranea*, named PIVL. Its structure consists of a monomeric chain that conserved all characteristics of Kunitz/BPTI-like domain and has a molecular mass of 7691.7 Da. Functionally, PIVL exhibits a potent anti-tumor cell effect without altering cell viability. Moreover, PIVL reduces human glioblastoma U87 cell adhesion, migration, and invasion by impairing the function of αvβ3 integrin [9]. Commonly, integrin dimers activate downstream oncogenic signaling pathways, especially the mitogen-activated protein kinase (MAPK) pathway and the phosphoinositide-3-kinase (PI3K)/AKT pathway in several cancer types, including glioblastoma [10]. Cilengitide, an RGD pentapeptide, was the first targeting integrin drug tested in clinical trials for treatment of glioblastoma patients [11]. GLPG0187 is another integrin antagonist agent that can be used for glioblastoma therapy [12]. On the other hand, we found that PIVL blocks angiogenesis by disturbing microtubule dynamics in transfected human mammary epithelial HMEC-1 cells and by reducing capillary-like structure on Matrigel^TM^ as well as neovascularization on chick chorioallantoic membrane [13]. These findings reveal that PIVL can have exquisite binding specificities and possesses a high potency for its targets, which are the main effectors in cancerogenesis process, making it an excellent therapeutic candidate. In this context, we resort to express a functional recombinant PIVL (rPIVL) and to inspect whether it preserves the same pharmacological properties of the native peptide to have an efficient and easier way for its production.

## 2. Results and Discussion

### 2.1. Amplification of the cDNA Encoding PIVL

**Figure 1:**
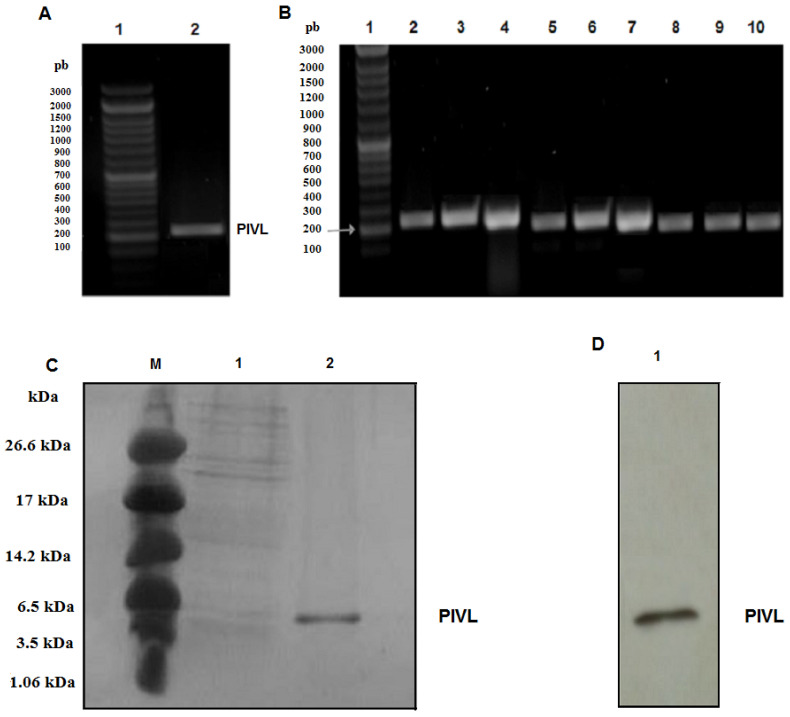
Expression and purification of rPIVL: (**A**) PCR amplification of PIVL cDNA. Lane 1: 100 bp DNA ladder; lane 2: amplified PIVL cDNA. (**B**) Colony PCR (gene-specific) screening of PIVL encoding region in pGEMT vector. Lane 1: 100 bp DNA ladder; lane’s 2–10: positive PIVL encoding clones. (**C**) Aliquot of purified protein was resolved by SDS-PAGE gel and stained with coomassie brilliant blue. Lane M: Ultra low Protein marker; lane 1: the flow through; lane 2: purified His-tag-rPIVL. (**D**) Western blot was probed with an anti-His-tag antibody. Lane 1 corresponds to purified rPIVL.

The cDNA encoding PIVL was cloned from the reverse-transcription PCR products obtained from total RNA of *Macrovipera lebetina transmediterranea* venom gland. Then, PCR amplification of PIVL cDNA was carried out using two primers bearing on their ends the appropriate *NdeI* (5′) and *XhoI* (3′) restriction sites. The PCR product mixture obtained after amplification was allowed to migrate on 1% agarose gel. As shown in Figure 1A, lane 2, a single band of cDNA coding PIVL, was migrated at the expected size around 213 bp. After the purification step, this PCR product mixture was put in a pGEMT vector and transformed in *E. coli* Top10 strains. Great deals of colonies were verified by PCR using the same primers and then sequenced (Figure 1B). PIVL cDNA is amplified in the distinct clones, and their sequence was found to be identical to the published one, i.e., the, cDNA coding PIVL with accession number HE800183 [9].

### 2.2. Expression and Purification of Recombinant PIVL

The recombinant constructs rPIVL-pET-22b (+) was introduced into *E. coli BL21* to be expressed in the form of a recombinant protein. It has been reported that *E. coli* become one of the microorganisms of choice for recombinant protein expression, especially for the production of Kunitz-type serine proteins from venoms and/or from the human blood fluke *Schistosoma mansoni*, such as Flavikunin and SmKI-1 [14,15]. As expected, a high yield of rPIVL expressed in *E. coli* BL21 (DE3) cells was obtained in this work. Indeed, the rPIVL yield value (10 mg/mL), estimated after purification with Ni^2+^ affinity chromatography, was equal to or greater than those reported in literature [14,15].

In our work, the ideal culture conditions of rPIVL expression were 1 mM of IPTG as inducer at a temperature of 37 °C and with 3 h as post-induction time (Supplemental Data, Figure S1). In these conditions, the rPIVL was highly expressed in *E. coli BL21* system, and it was accumulated as insoluble protein in inclusion bodies that were easily purified. For that, following cell lysis, the insoluble protein were harvested and solubilized in the denaturing buffer containing urea. The unfolded rPIVL was recovered following the different stages described in the Materials and Methods section. After that, the rPIVL was purified by Ni^2+^ affinity chromatography. Six His-tags are generally utilized for the purification of recombinant proteins by affinity chromatography for several reasons, such as the lower molecular mass and for conserving the biological activity. The rPIVL was eluted by 100 mM imidazole. It was then collected and loaded to SDS-PAGE. A single band with a molecular mass of about 8 kDa corresponding to rPIVL was detected (Figure 1C, lane 2). The expression of rPIVL was then verified by immunoblot using an anti-His antibody as shown in Figure 1D, lane 1.

### 2.3. Recombinant PIVL Affects U87 Cells Adhesion and Motility

**Figure 2:**
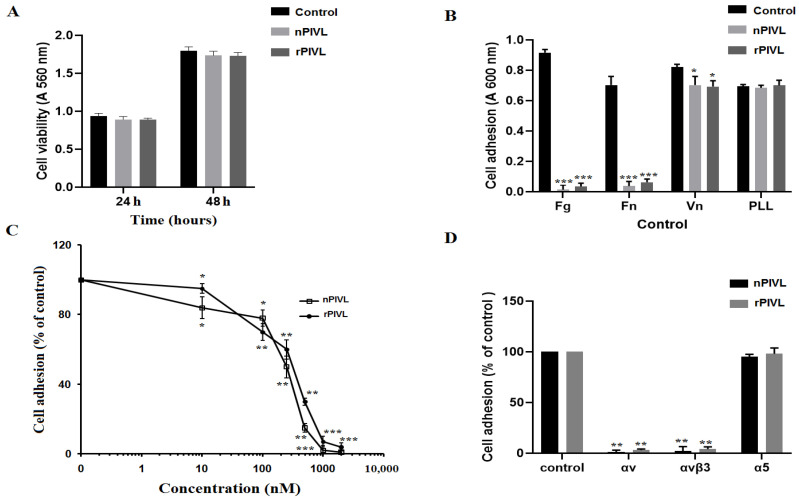
Recombinant PIVL enhances human glioblastoma U87 cells adherence: (**A**) Cells were cultured in the absence (black bars) or in the presence of 1 µM nPIVL (light grey bars) or rPIVL (dark grey bars) for 24 and 48 h to evaluate cell viability by MTT assay. (**B**) Cells were incubated without (black bars) and with 1 μM nPIVL (light grey bars) or rPIVL (dark grey bars) for 30 min. Cells were then allowed to attach on fibrinogen (Fg), fibronectin (Fn), vitronectin (Vn), or poly-L-lysine (PLL). (**C**) Cells seeded on wells coated with fibrinogen were incubated with various concentrations of nPIVL (open squares) or rPIVL (closed circles). (**D**) U87 cells were incubated in the absence or in the presence of various antibodies against αv, αvβ3, or α5 and allowed to attach on microtiter plates coated with nPIVL (black bars) or rPIVL (grey bars). Data are representative of three independent experiments performed in triplicate, expressed as mean (±SD). *p* < 0.05 was considered statistically significant and is indicated with asterisks over the value (* *p* < 0.05, ** *p* < 0.01, *** *p* < 0.001).

The key role of cell adhesion in cancer progress is well documented. It emerges that cells lose their primary site, move through the extracellular matrix (ECM), extravasate healthy tissue, and form distant metastasis. This knowledge can serve as optimistic approaches to cancer treatment by targeting cell adhesion molecules [16]. In this work, we found that neither native PIVL (nPIVL) nor rPIVL affects cell viability (Figure 2A). As shown in Figure 2B, both rPIVL and nPIVL at 1 μM notably blocked the adhesion of U87 cells on fibrinogen and fibronectin, while no effect was observed neither on vitronectin nor on poly-L-lysine (an integrin independent substratum). Accordingly, we then demonstrate that rPIVL efficiently inhibited the adhesion of U87 on fibrinogen with a concentration-dependent manner. The IC_50_ values of nPIVL and rPIVL are of 250 nM and 350 nM, respectively (Figure 2C). This result indicates that the recombinant and the native peptide have nearly the same activity, showing that the rPIVL is as active as the native one.

We previously reported that the anti-adhesive effect of nPIVL is due to its interaction with the integrin αvβ3 as adhesion receptor in U87 cells [9]. Therefore, the rPIVL was tested on the function-blocking antibodies against integrins. As shown in Figure 2D, rPIVL abolishes U87 cell adhesion on monoclonal antibodies against αv and αvβ3 integrins.

**Figure 3:**
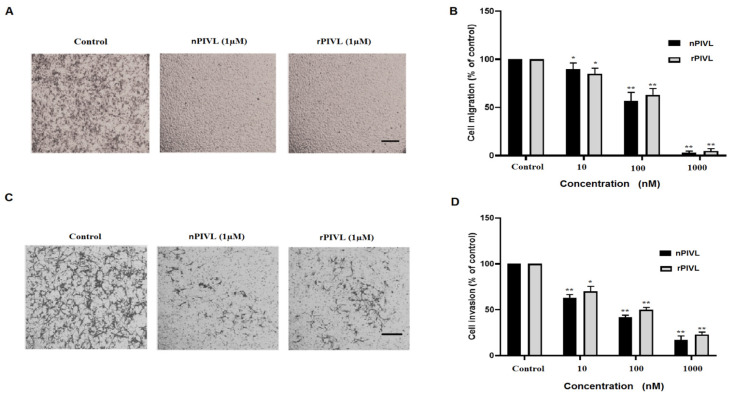
Recombinant PIVL abolishes human glioblastoma motility and invasion (**A**) Cells were treated for 30 min with 1 μM nPIVL or rPIVL and allowed to migrate through the porous filter. Scale bar: 100 μm. (**B**) Histogram presented cell migration towards fibrinogen treated with various concentrations of nPIVL (black bars) or rPIVL (grey bars). (**C**) Cells were incubated without or with 1 μM nPIVL or rPIVL for 30 min. Cells invaded the layer of Matrigel™ in modified Boyden chamber. Scale bar: 100 μm. (**D**) Histogram represented concentration-effect of nPIVL (black bars) or rPIVL (grey bars) on U87 cell invasion. Data are representative of three independent experiments performed in triplicate expressed as mean (±SD). *p* < 0.05 was considered statistically significant and is indicated with asterisks over the value (* *p* < 0.05, ** *p* < 0.01).

Glioblastoma cells are essentially characterized by rapid migration and high power to invade adjacent healthy brain tissue. This phenomenon is responsible for the worst prognostic and poor survival for patients [17]. Thus, stopping or reducing glioblastoma infiltration is of great importance for developing novel therapeutic approaches to treat glioblastoma patients. Accordingly, we first examined the effect of rPIVL and nPIVL on U87 cell migration towards fibrinogen by Haptotaxis assay assessed in modified Boyden chambers. As shown in Figure 3A, both nPIVL and rPIVL notably impaired migration of U87 cells on fibrinogen. The IC_50_ values were of 200 nM and 300 nM, respectively, and the maximal effect was obtained at 1 μM (Figure 3B).

In a second step, we evaluated the anti-invasive effect of both native and recombinant molecules on U87 cells and their capacity to infiltrate the layer of matrigel added in the upper side wells of modified Boyden chambers. As demonstrated in Figure 3C, rPIVL as well as nPIVL significantly reduced the invasion of human glioblastoma U87 cells in a concentration-dependent manner. The maximal effect is about approximately 80% and 70% at 1 µM of nPIVL and rPIVL, respectively (Figure 3D).

Thus, we report here a functional recombinant PIVL (rPIVL) with potent anti-tumor cell effect. As far as we know, the only reported functional expressed peptide homologous to Kunitz-domain was dendrotoxin K from black mamba, which inhibits voltage-sensitive neuronal K+ channels [18,19]. In order to verify whether the ion channels are involved in the anti-tumor cell effects of PIVL, we incubated U87 cells with tetraethylammonium (TEA), a K+ channel blocker. We demonstrate that PIVL completely inhibits cells adhesion even in presence of TEA (Supplemental Data, Figure S2), showing that PIVL did not interfere with K+ channels to block U87 cell adhesion. This suggests that PIVL has different mechanism of action than dendrotoxins.

### 2.4. Recombinant PIVL Blocks Angiogenesis

**Figure 4:**
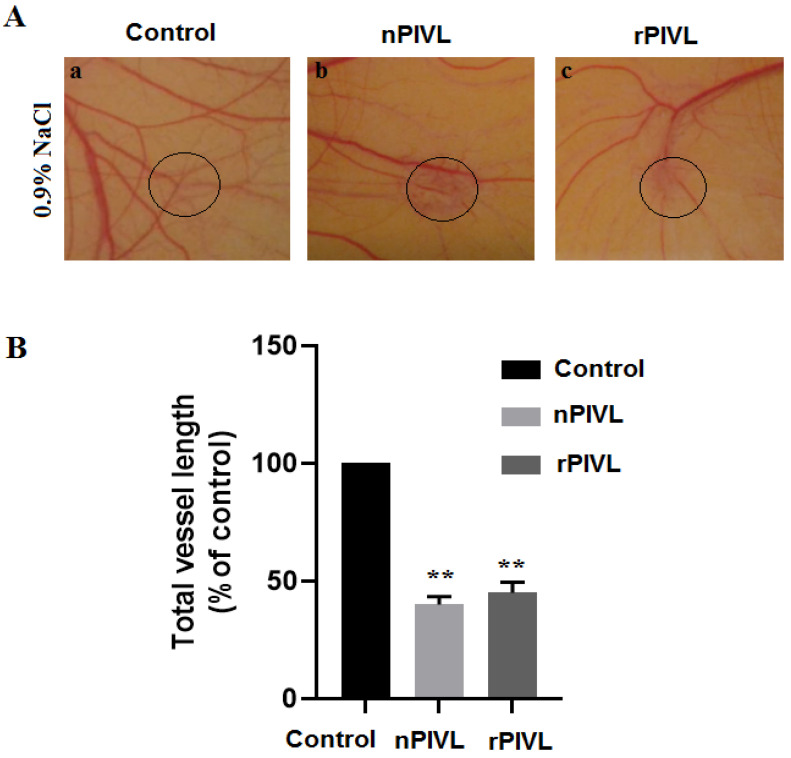
Recombinant PIVL blocks angiogenesis on CAM model (**A**) After incubation for 48 h in the absence or in the presence of 1 µM of nPIVL or rPIVL, neovascularization was photographed using a digital camera. (**B**) Quantification and measurement of total vessel length were assessed on 50% of the total chicken chorioallantoic membrane area. The experiment was done three times using four CAMs in each group. *p* < 0.05 was considered statistically significant and is indicated with asterisks over the value (** *p* < 0.01).

To further characterize the rPIVL properties, an ex-vivo model of angiogenesis was assessed using chick chorioallantoic membrane (CAM) assays, as previously described [13]. After dissection and incubation of chick embryos in humidified incubator, filter discs soaked with 0.9% of saline solution or with nPIVL/rPIVL at 1 µM concentration were applied on the chick chorioallantoic membrane. As shown in Figure 4A, rPIVL as well as nPIVL decreased neovascular density after 48 h. Furthermore, both rPIVL and nPIVL did not affect pre-existing capillaries and branching vessels. The measure of total vessel length, using the software program ImageJ, showed a reduction by 55% and 60%, respectively, compared with the control (Figure 4B).

### 2.5. Recombinant and Native PIVL Modulate PI3/AKT and MAPK Signaling Pathways in Glioblastoma Cells

**Figure 5:**
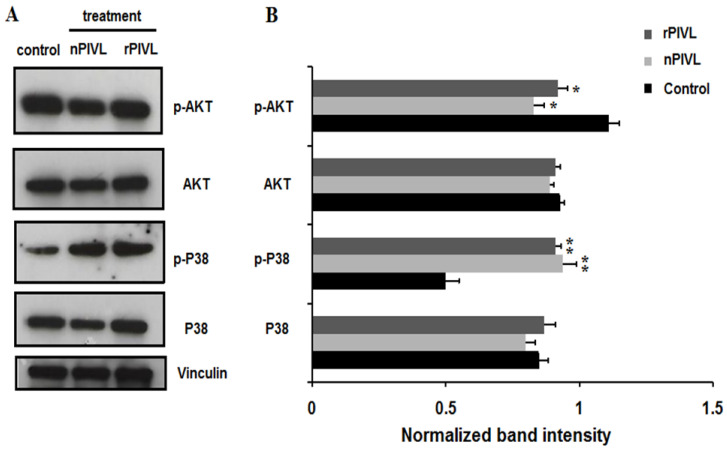
Recombinant PIVL and native PIVL modulate signaling pathways on glioblastoma cells. (**A**) Western blot analysis was performed to detect phosphorylated AKT and P38 in the presence or absence of nPIVL or rPIVL at 1 μM each for 24 h. (**B**) Quantification of phosphorylated and total signaling proteins was performed by using the software program ImageJ. The results are expressed as means ± s.e.m (standard error of the mean) of three independent Western blot experiments. *p* < 0.05 was considered statistically significant and is indicated with asterisks over the value (* *p* < 0.05, ** *p* < 0.01).

As previously demonstrated, we confirmed that nPIVL exhibited potent anti-tumor cell properties when tested on human glioblastoma U87 cells by interfering with αvβ3 integrin [9]. To explore the effect of nPIVL and rPIVL on signaling pathways, we performed a Western blot against AKT and P38 using specific antibodies. As shown in Figure 5, we found that rPIVL as well as nPIVL reduced expression of phosphorylated AKT, associated with an increase of phosphorylated P38 MAPK. Accumulating evidences indicate that activated Akt affects critical cellular and physiological processes, such as cellular growth, cell survival, apoptosis, migration, invasion, and angiogenesis [20]. The p38 mitogen-activated protein kinase (MAPK) pathway integrates various types of stress signals and mediates anoikis, a programmed cell death, due to a lack of cell attachment to extracellular matrix (ECM) [21]. Besides, an essential role of p38 MAPK in modulation of cell migration and invasion, in which integrins play a critical role, has been reported [22,23]. Thus, activating p38 MAPK could be a novel strategy to control human malignancies. Altogether, our results suggest that rPIVL as well as nPIVL interferes with PI3/AKT and MAPK pathways by reducing the levels of phosphorylated AKT and increasing expression of phosphorylated P38, thereby affecting adhesion, migration, and invasion of U87 glioblastoma cells.

## 3. Conclusions

The elucidation of oncogenic signaling pathways activated in many cancer types, including glioblastoma, requires research of new agents and identification of specific target molecules in neoplastic cells. We report in this work the first expressed snake venom Kunitz-type protease inhibitor that exhibits potent anti-tumor cell effects and anti-angiogenic properties. Interestingly, we found similar functional characteristics between recombinant PIVL and the native peptide. Targeting MAPK and PI3/AKT pathways by both native and recombinant PIVL will undoubtedly promise for a new generation of anticancer drugs. Further structure-function correlation studies could be henceforth performed by site-directed mutagenesis of the rPIVL protease active sequence to prevent possible side effects and will be a challenging task for the development of recombinant peptide drugs.

## 4. Materials and Methods

### 4.1. Materials, Reagents, and Ethics Statement

Venom was collected by the veterinarian service of the Pasteur Institute of Tunis (Tunis, Tunisia). Experiments were carried out following the European Community Council Directive (2010/63/EU) for experimental animal care and procedures. Bacteria strains were cultured on Luria–Bertani (LB) medium (Invitrogen) in the presence of ampicilin at 100 μg/mL. PCR Purification DNA and Gel Extraction Kit, *NdeI*, *XhoI*, and ligase enzymes were obtained from promega. Urea and L-cysteine were acquired from Sigma (St. Quentin Fallavier, France). Isopropyl β-D-1-thiogalactopyranoside (IPTG) and antibiotic selection (Ampicilin) were obtained from Invitrogen (Villebon, France).

### 4.2. Host Strains and Plasmids

For cloning and protein expression of recombinant PIVL, both vectors were used in this work, pGEMT and pET-22b (+), respectively. *E. coli* Top10 and *BL21* (DE3) pLysS competent cells (Novagen) were employed in PIVL cDNA cloning. For expression, the amplified PIVL cDNA was cloned between the *NdeI* and *XhoI* restriction sites in the pET-22b (+) with a C-terminal poly-His-tag.

### 4.3. Molecular Cloning Procedure of PIVL

The purification and the structural characterizations of PIVL were performed as described [9]. Total RNA was extracted from a single venom gland of *Macrovipera lebetina transmediterranea*. Venom gland was homogenized in liquid nitrogen, and total RNA was extracted using guanidinium thiocyanate/phenol/chloroform as described [24]. The cDNA encoding the total sequence of PIVL was obtained by reverse transcription (RT) using Moloney Murine Leukemia Virus Reverse Transcriptase (M-MLV RT) according to instruction conditions described in [24].

The cDNA corresponding to PIVL was amplified by PCR using Pfu polymerase (Promega, Madison, WI, USA) and a specific primer incorporating their upstream- and downstream-appropriate *NdeI* and *XhoI* restriction sites (in bold). The sequences of the forward and reverse primer were, respectively: F1 5′-**CATATG**CAGGACCGTCCAAAGTTTTGT-3′ and R1 5′-**CTCGAG**TCGGGGTTGTATCCCCTTTCT-3′. The following conditions were the initial denaturation step at 95 °C for 30 s, then 30 cycles of denaturation at 95 °C for 30 s, hybridization at 55 °C for 30 s, and extension at 72 °C for 30 s; the final step was the extension at 72 °C for 7 min. The amplified PIVL cDNA (213 bp) was purified and cloned in pGEM-T vector (Promega, Madison, WI, USA). A total of 2.5 μL of ligation mixture (containing 10–20 ng vector DNA) was used for *E. coli* Top10 competent cells transformation. Finally, the rPIVL-pGEMT construction was confirmed by colony PCR, restriction analysis, and sequencing.

### 4.4. Expression of Recombinant PIVL

The cDNA corresponding to PIVL was amplified by PCR using Pfu polymerase (Promega, Madison, WI, USA), the rPIVL-pGEMT construction was used as template DNA and a specific primer described previously. The amplified product was purified and put in expression vector pET-22b (+). The recombinant plasmids were transformed in *E. coli* BL21 (DE3), and the transformed cells were grown on LB agar plates with ampicilin at 100 μg/mL. The screening step of the recombinant *E. coli* strains was carried out by colony PCR and sequencing.

To optimize the conditions of the PIVL protein expression, single colony was taken during 4 h at 37 °C in 2 mL of LB Broth medium containing 100 μg/mL ampicilin. The PIVL protein expression was induced by various concentrations of IPTG (0.4, 1, and 2 mM) with the optical density at 600 nm until it reached approximately 0.8. After induction, the induced cultures were shaken at 37 °C for one more 5 h. Aliquot samples (200 μL) were carried out for each culture every hour and centrifuged at 12,000× *g* rpm for 10 min. The pellet was resuspended in 0.1 mL of a solution containing 4% SDS, 100 mM Tris-HCl (pH 7), and 0.4 mM β-mercaptoethanol and analyzed in SDS-PAGE. For extensive rPIVL expression, the preculture (2 mL) was utilized to inoculate 250 mL LB Broth medium containing ampicillin at 100 μg/mL. The rPIVL expression in *E. coli* BL21 (DE3) was induced by addition the optimal concentration of IPTG at 1 mM. Induced cells were collected by centrifugation at 12,000× *g* rpm for 15 min, and the cell pellets were stored at −20 °C until the protein extraction was complete.

### 4.5. Protein Recovery and Purification

In order to purify rPIVL, *E. coli* BL21 strains were lysed in buffer solution (25 mM Tris-HCl, pH 8.0; 20 mM EDTA and 50 mM NaCl) for 35 min on ice, accompanied by sonication and centrifugation steps at 12,000× *g* rpm for 20 min. The expressed rPIVL protein was not in soluble form (supernatant), but it was detected in the inclusion bodies. For that, unfolded proteins were dissolved in denaturing buffer (25 mM Tris-HCl, pH 8; 8 M urea and 5 mM L-cysteine) by agitation overnight at 4 °C. The denatured rPIVL was recovered by dialyzing against 2 L of the buffer containing 25 mM Tris-HCl, pH 8; 0.8 M urea, and 5 mM L-cysteine overnight at 4 °C. A filtration process by the use of a filter (0.45 μm) was achieved. After that, the urea was removed from the recovered rPIVL protein by dialyzing one more against 2 L of the buffer (25 mM Tris-HCl pH 8.0, 10 mM NaCl) overnight at 4 °C, followed by centrifugation step at 12,000× *g* rpm for 35 min at 4 °C.

The obtained rPIVL proteins were loaded onto Nickel columns, stabilized previously with buffer containing 20 mM Tris-base pH 8.0, 320 mM NaCl, and 10 mM imidazole. After that, the Nickel column was washed with the identical buffer. The elution step of the rPIVL protein was carried out with elution buffer (20 mM Tris-base pH 8.0, 320 mM NaCl, and 100 mM imidazole), followed by a dialyzing step with the same buffer described above but without imidazole. The concentration of purified recombinant PIVL was determined by Bradford’s method.

### 4.6. Protein Electrophoresis and Western Blot Analysis

Protein expression of the rPIVL and the signaling pathway on human glioblastoma U87 cells were evaluated by Western blot. The purified rPIVL protein was separated in SDS-PAGE (15%) by electrophoretic buffer (25 mM Tris; 250 mM Glycine; 1‰ SDS) for 2 h. After SDS-PAGE, the purified rPIVL were electroblotting onto a PVDF (polyvinylidene difluoride, Thermo Fisher Scientific, Waltham, MA, USA) membrane in transfer buffer (39 mM Glycine; 48 mM Tris; 0.037% SDS; Methanol 20%) for 1 h at constant voltage (120 V). The membrane was saturated to prevent non-specific binding for 1 h at ambient temperature with 5% skim milk in PBS-T (0.9% NaCl in 10 mM phosphate buffer, pH 7.4, and 0.1% Tween-20). The primary antibody anti-His from Sigma diluted to 1:5000 was used, followed by the Horseradish Peroxidase (HRP) secondary antibody at the same concentration. The ECL Western Blotting Detection Reagent (Amersham Pharmacia Biotech, Piscataway, NJ, USA) was used to detect peroxidase activity.

To check the phosphorylation levels of AKT and P38, protein samples were loaded (20 μg/lane), separated by electrophoresis method, and transferred on PVDF membrane following the same protocol as described above. Primary antibodies used for analysis, including rabbit monoclonal anti-phospho-p38 MAPK (Thr180/Tyr182), rabbit monoclonal anti-phospho-Akt (Serine 473), rabbit monoclonal anti-Akt1, rabbit monoclonal p38 MAPK, and anti-vinculin antibody along with the HRP-conjugated secondary antibodies, were purchased from Cell Signalling Technology (Danvers, MA, USA).

### 4.7. Cell Culture and Adhesion Assays

U87 human glioblastoma cells were prepared in MEM media with 10% FCS. As previously described for adhesion assays [25,26], cells were seeded to 96-well plate coated with 10 μg/mL for fibronectin (Fn), vitronectin (Vn), and poly-L-lysine (PLL) and 50 µg/mL for fibrinogen (Fg) during 1 h at 37 °C. Then, cells were fixed and colored by 0.1% crystal violet. The absorbance values were obtained at 600 nm. The half maximal inhibitory concentration (IC 50) values were calculated using Graphpad Prism Software. Adhesion assay on antibodies was assessed on various antibodies against αv, αvβ3, or α5 and allowed to attach on microtiter plates coated with 10 μg/mL nPIVL or 10 μg/mL rPIVL as described previously [9]. Adhesion assay with ion channel blocker was performed by incubated U87 with 10^−6^ M of tetraethylammonium (TEA) for 1 h. Then cells were treated in absence or in presence of 1 µM of PIVL. Quantification of adhesion was assessed as described previously.

### 4.8. Cell Viability Assays

Cell viability was performed by MTT (3-(4,5-dimethylthiazol-2-yl)-2,5-diphenyltetrazolium bromide) assay [27]. Briefly, 500 μg/mL of MTT were added to the cell culture for 3 h. After production of formazan crystals, 100 μL of DMSO were added to dissolve purple precipitate, and absorbance was measured at 560 nm.

### 4.9. Cell Migration Assays

Haptotaxis assays were assessed in modified Boyden chambers purchased from NeuroProbe Inc., Bethesda, MD, USA. Cells were treated without or with 1 µM of nPIVL or rPIVL and allowed to migrate on specific membranes coated with 50 μg/mL of fibrinogen for 5 h at 37 °C as described [28]. Migrated cells through the porous membrane were fixed and colored with 0.1% crystal violet. The absorbance was then measured at 600 nm, and the half maximal inhibitory concentration (IC 50) values were calculated using Graphpad Prism Software as previously described [25,29].

### 4.10. Cell Invasion Assays

Cell invasion assays were assessed in modified Boyden chambers coated with 20 µL of diluted Matrigel™ (BD Biosciences, Pont de Claix, France). In the first step, cells were treated without or with various concentrations of nPIVL or rPIVL and allowed to invade the layer of Matrigel™ for 24 h. In the second step, cells were fixed and stained with 0.1% crystal violet, and absorbance was measured at 600 nm as previously described [9].

### 4.11. Chicken Chorioallantoic Membrane Assay

An ex-vivo model for angiogenesis was performed using chick embryos from 3-day-old eggs dissected in Petri dishes and placed in humidified incubator at 37 °C. Five days later, filter disks were soaked in 0.9% saline solution and 1 µM of nPIVL or rPIVL and applied on the chicken chorioallantoic membrane (CAM). Neovascularizations were photographed with a digital camera at 10× magnification, and quantification was assessed using the software program ImageJ as described [13].

### 4.12. Statistical Analysis

Data represent a representative experiment (from three independent experiments) expressed as mean ± standard deviation (SD). The statistical significance of differential findings between experimental and control groups was determined by Student’s *t*-test using GraphPad Prism 4 software, and *p* < 0.05 was considered statistically significant.

## Data Availability

Not applicable.

## Supplementary Materials

The following supporting information can be downloaded at: https://www.mdpi.com/article/10.3390/toxins14030170/s1, Figure S1: Optimization of rPIVL expression: Samples from different culture steps were separated by SDS-PAGE. Performed parameters of expression were carried out, such as the IPTG concentrations (0.4, 1 and 2 mM) and post-induction time (1–5 h). Lane M: Ultra low Protein marker; -: control, non-induced culture; Figure S2: Effect of PIVL on U87 cells incubated with ion channel blocker: Cells were seeded and incubated with tetraethylammonium (TEA). Adhesion cells were performed in presence or in absence of nPIVL.
