# Supplementary material for: Expression of the First Recombinant Anti-Tumoral Snake Venom Kunitz-Type Serine Protease Inhibitor
Source: Toxins (Basel). 2022 Feb 25;14(3):170. doi: 10.3390/toxins14030170 (PMC8955015; doi:10.3390/toxins14030170)
Supplement: Supplementary file 1 [file toxins-14-00170-s001.zip › toxins-1521710-supplementary.pdf]

toxins

MDPI

Article

Expression of the First Recombinant Anti-Tumoral Snake Venom Kunitz-Type Serine Protease Inhibitor

Maram Morjen, Wassim Moslah, Imen Touihri-Baraketi, Najet Srairi-Abid, José Luis, Naziha Marrakchi, and Jed Jebali

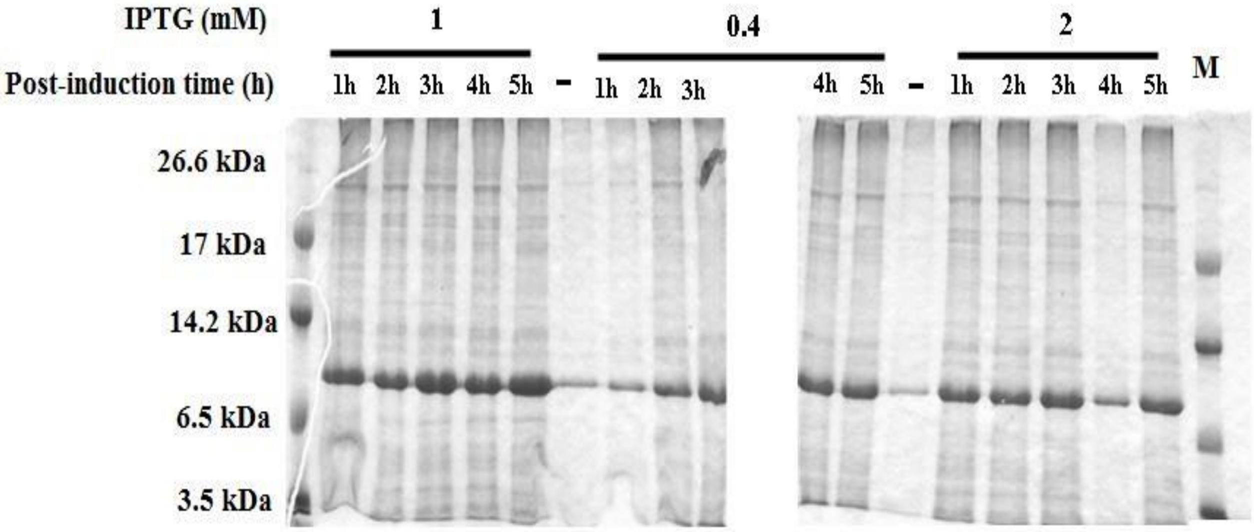

Figure S1. Optimization of rPIVL expression: Samples from different culture steps were separated by SDS-PAGE. Performed parameters of expression were carried out, such as the IPTG concentrations (0.4, 1 and 2 mM) and post-induction time (1 h-5 h). Lane M: Ultra low Protein marker; - : control, non-induced culture.

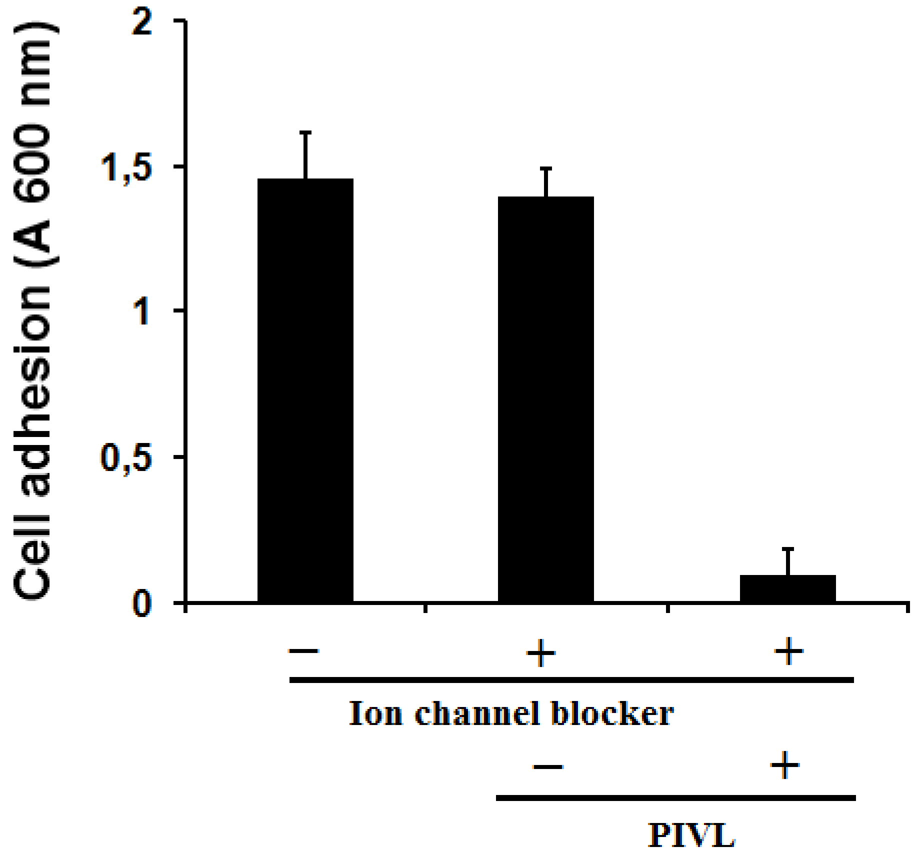

Figure S2. Effect of PIVL on U87 cells incubated with ion channel blocker: Cells were seeded and incubated with tetraethylammonium (TEA). Adhesion cells were performed in presence or in absence of nPIVL.
